# Multimodal robot-assisted English writing guidance and error correction with reinforcement learning

**DOI:** 10.3389/fnbot.2024.1483131

**Published:** 2024-11-20

**Authors:** Ni Wang

**Affiliations:** School of Humanities and International Education, Xi'an Peihua University, Xi'an, Shaanxi, China

**Keywords:** VVG19, ALBEF, English text generation, reinforcement learning, multimodal robot

## Abstract

**Introduction:**

With the development of globalization and the increasing importance of English in international communication, effectively improving English writing skills has become a key focus in language learning. Traditional methods for English writing guidance and error correction have predominantly relied on rule-based approaches or statistical models, such as conventional language models and basic machine learning algorithms. While these methods can aid learners in improving their writing quality to some extent, they often suffer from limitations such as inflexibility, insufficient contextual understanding, and an inability to handle multimodal information. These shortcomings restrict their effectiveness in more complex linguistic environments.

**Methods:**

To address these challenges, this study introduces ETG-ALtrans, a multimodal robot-assisted English writing guidance and error correction technology based on an improved ALBEF model and VGG19 architecture, enhanced by reinforcement learning. The approach leverages VGG19 to extract visual features and integrates them with the ALBEF model, achieving precise alignment and fusion of images and text. This enhances the model's ability to comprehend context. Furthermore, by incorporating reinforcement learning, the model can adaptively refine its correction strategies, thereby optimizing the effectiveness of writing guidance.

**Results and discussion:**

Experimental results demonstrate that the proposed ETG-ALtrans method significantly improves the accuracy of English writing error correction and the intelligence level of writing guidance in multimodal data scenarios. Compared to traditional methods, this approach not only enhances the precision of writing suggestions but also better caters to the personalized needs of learners, thereby effectively improving their writing skills. This research is of significant importance in the field of language learning technology and offers new perspectives and methodologies for the development of future English writing assistance tools.

## 1 Introduction

In the current field of natural language processing, English Text Generation technology is becoming increasingly important. Firstly, this technology not only enhances machines' understanding and generation of language but also advances automated content creation. For example, it plays a crucial role in news reporting, advertising copy, and literary creation. Secondly, with the continuous development of artificial intelligence technology, English Text Generation not only provides more natural and fluent communication but also meets personalized and context-specific needs, thereby improving user experience. Overall, this technology not only helps to improve the performance of language models but also holds broad application prospects in fields such as education and entertainment. Therefore, research into this technology is of significant practical importance and far-reaching impact.

Traditional methods for English text generation primarily rely on symbolic AI and knowledge representation. During this phase, expert systems, as a classic technology, generate text by utilizing predefined rules and knowledge bases. The main advantage of this approach is its ability to provide high-precision semantic processing, ensuring that the generated text adheres to specific knowledge and rules (Liu A. et al., 2021). Another method is rule-based text generation, which relies on a systematic set of language rules to ensure that the generated text is consistent and standardized in grammar and structure (Gašpar et al., 2023). Additionally, manual feature extraction is a commonly used technique, where features are manually selected and defined to drive text generation, allowing the model to focus on key language features and improve the quality of the generated text (Wang et al., 2020). These methods have distinct advantages in their respective application domains, such as high control, good interpretability, and strong structural capabilities. However, they also have certain shortcomings. For example, expert systems and rule-based methods often lack flexibility when dealing with complex and dynamic language environments. Although manual feature extraction can capture important features, it often struggles to adapt to language changes and diversity. Therefore, these traditional methods need further improvement and expansion to meet modern demands.

To address the shortcomings of traditional algorithms in terms of flexibility and adaptability, data-driven and machine learning-based algorithms have been widely applied in English text generation. These methods primarily generate text by automatically learning language patterns and features from large amounts of data. This approach has strong adaptive capabilities, allowing it to handle complex language structures and diverse expressions (Zeng, 2016). For example, decision tree-based algorithms effectively handle classification and regression problems by recursively partitioning datasets to form a series of rules. Random forest-based methods further enhance text generation stability and accuracy by constructing an ensemble model of multiple decision trees, demonstrating exceptional performance, particularly in handling high-dimensional data (Jalal et al., 2022). Additionally, the multilayer perceptron, as a type of feedforward neural network, captures complex relationships and deep features in language through the nonlinear combination of multiple hidden layers, generating more natural and fluent text (Sewunetie and Kovács, 2022). However, these methods have the drawbacks of high training complexity and a strong dependence on large-scale data, and they often exhibit insufficient generalization performance when dealing with extreme or rare language patterns.

To address the shortcomings of statistical and machine learning algorithms in feature extraction and model generalization, deep learning-based algorithms have been widely applied in English text generation. These methods primarily generate more natural and high-quality text by automatically learning complex language features and patterns through deep neural networks. This approach has significant advantages, such as the ability to handle large amounts of unstructured data, capture complex dependencies in language, and generate highly coherent and contextually appropriate content. For instance, Convolutional Neural Networks (CNNs) effectively process structural information in sentences or paragraphs by extracting local features of the text (Uchendu et al., 2020). Generative Adversarial Networks (GANs), through adversarial training between a generator and a discriminator, can generate content that closely resembles real text, enhancing the diversity and creativity of text generation (Chang et al., 2023). The Transformer model, with its self-attention mechanism, significantly improves the efficiency and accuracy of text generation, particularly excelling in the generation of long texts (Phan et al., 2022). The attention mechanism further strengthens the model's ability to capture contextual information, making the generated text more coherent and semantically consistent (Liu Y. et al., 2021). However, these methods have drawbacks, such as high model complexity, significant computational resource demands, and insufficient robustness when handling rare or unseen data.

To address the challenges posed by deep learning methods in English Text Generation, such as high model complexity, significant computational resource demands, and insufficient robustness when handling rare or unseen data, we propose a method named ETG-ALtrans. This method is based on an improved ALBEF (Align before Fuse) model and is applied to English writing guidance and error correction technology assisted by a multimodal robot. The traditional ALBEF model primarily aligns and fuses visual and linguistic information to handle multimodal tasks but faces limitations in complex language generation and semantic understanding. To overcome these issues, we optimized the ALBEF model to enhance its ability to capture contextual information in text generation while reducing its dependency on computational resources. ETG-ALtrans integrates multimodal information such as text, images, and speech to provide comprehensive English writing guidance. It effectively identifies and corrects grammatical and semantic errors in writing and generates more natural and fluent text based on context. Additionally, our method demonstrates stronger robustness when dealing with rare and unseen language patterns, improving the model's adaptability in diverse application scenarios. Experimental validation shows that ETG-ALtrans outperforms on multiple metrics, offering new insights into the development of English writing guidance technology.

- ETG-ALtrans introduces an improved ALBEF model, which combines multi-modal information to improve the comprehensive understanding and generation capabilities of text and visual content.
- This method is adaptable to multiple scenarios, efficiently handles complex writing tasks, has strong versatility, and is suitable for a variety of English writing and error correction scenarios.
- Experimental results show that ETG-ALtrans is significantly better than traditional methods in accuracy, fluency and grammatical standardization, improving the overall effect of English writing guidance and error correction.

## 2 Related work

### 2.1 Text generation

Text generation technology is a key research area in natural language processing (NLP), aiming to automatically generate natural language text that adheres to grammatical, semantic, and contextual requirements. Early text generation techniques relied primarily on template or rule-based methods. While these methods performed well in specific scenarios, they lacked flexibility and contextual understanding, making them less suitable for complex language generation tasks (Lin et al., 2024b). With the advent of statistical language models, particularly *n*-gram models, text generation gradually shifted toward data-driven approaches. In recent years, neural networks, especially Recurrent Neural Networks (RNNs) and Long Short-Term Memory networks (LSTMs), have played a significant role in text generation. These models can capture sequential information in text, resulting in more fluent and coherent sentences. However, these models also face challenges in handling long-range dependencies (Wang et al., 2019b). The introduction of Transformer models has brought a breakthrough in text generation technology. The self-attention mechanism of Transformers can better handle long-range dependencies and significantly improve the quality and efficiency of text generation. Transformer-based pre-trained models, such as the GPT series and BERT, have become mainstream in the field of text generation. These models, through large-scale pre-training and fine-tuning, can generate high-quality text for various tasks (Yuan et al., 2021). Notably, GPT models are widely used in dialogue systems, content creation, code generation, and other areas due to their exceptional generation capabilities. However, text generation still faces challenges such as controllability, diversity, coherence, and reducing bias and ethical issues. Future research directions may include more efficient generation models, better model interpretability, and real-time quality assessment and control of generated content.

### 2.2 Convolutional neural networks

Convolutional Neural Networks (CNNs) have become a core technology in computer vision since their breakthrough in image recognition tasks in 2012. CNNs are characterized by local connections, shared weights, and pooling operations, which give them strong feature extraction capabilities for handling two-dimensional data like images. Beyond image recognition, CNNs are widely applied in other visual tasks such as object detection, image segmentation, and image generation (Lin et al., 2024a). For example, in object detection, Faster R-CNN significantly improves detection speed and accuracy by introducing a Region Proposal Network (RPN). In image segmentation, architectures like U-Net and SegNet achieve fine-grained semantic segmentation by classifying each pixel in the image (Wang et al., 2019a). In addition to computer vision, CNNs are increasingly applied in other fields. In natural language processing, CNNs are used for text classification, sentiment analysis, and more. By converting text into matrix form, CNNs can capture local features of text and achieve efficient classification. In bioinformatics, CNNs are used for analyzing gene sequences and predicting protein structures, effectively identifying important patterns and features in biological sequence data. Furthermore, CNNs are applied in signal processing and time-series analysis, where convolution operations on one-dimensional or multidimensional data help analyze complex signals effectively (Fishel and Loeb, 2012). Despite the strong performance of CNNs across various fields, there are some limitations, such as reliance on large amounts of labeled data and the need for fine-tuning model structures and parameters. Future research directions may include more efficient model architectures, semi-supervised or unsupervised learning methods to reduce labeling requirements, and model optimization in low-computation resource environments (De Angelis et al., 2023).

### 2.3 Multimodal technology

Multimodal technology refers to the processing and understanding of information by combining different types of data (e.g., text, images, audio, video). With the diversification of data forms and advancements in computational capabilities, the importance of multimodal technology in artificial intelligence has increasingly been recognized. Early multimodal technologies focused on simple feature fusion and joint modeling, such as concatenating or averaging image and text features to achieve multimodal information integration. However, these methods often struggled to capture complex relationships between different modalities, leading to poor performance in handling multimodal data (Wang et al., 2016). Recent advancements in deep learning have significantly progressed multimodal technology. Neural network-based multimodal models, such as those combining Convolutional Neural Networks (CNNs) for image processing, Recurrent Neural Networks (RNNs) for text processing, and fully connected layers for fusion, have become mainstream. These models effectively integrate multimodal information while maintaining the independence of each modality's features, thereby improving overall task performance. The introduction of Transformer models has further advanced multimodal technology, achieving breakthroughs in handling long-range dependencies and cross-modal alignment. Models like ALBEF (Align Before Fuse) enhance the complementarity and synergy of multimodal information by aligning modalities before fusion (Fishel and Loeb, 2012). Multimodal technology has found extensive applications in various scenarios, such as image-text retrieval, cross-modal translation, and video description generation. In healthcare, multimodal technology combines medical images and text reports for more accurate disease diagnosis and treatment recommendations. In autonomous driving, multimodal technology integrates data from cameras, radar, and LiDAR to enhance environmental perception and decision-making capabilities (Qushem et al., 2021). However, multimodal technology still faces challenges such as data heterogeneity, modality misalignment, and modality weight allocation. Future developments may include more effective multimodal alignment and fusion strategies, more interpretable and robust multimodal models, and efficient deployment and optimization of multimodal systems in practical applications.

## 3 Methodology

### 3.1 Overview of our network

**Figure 1:**
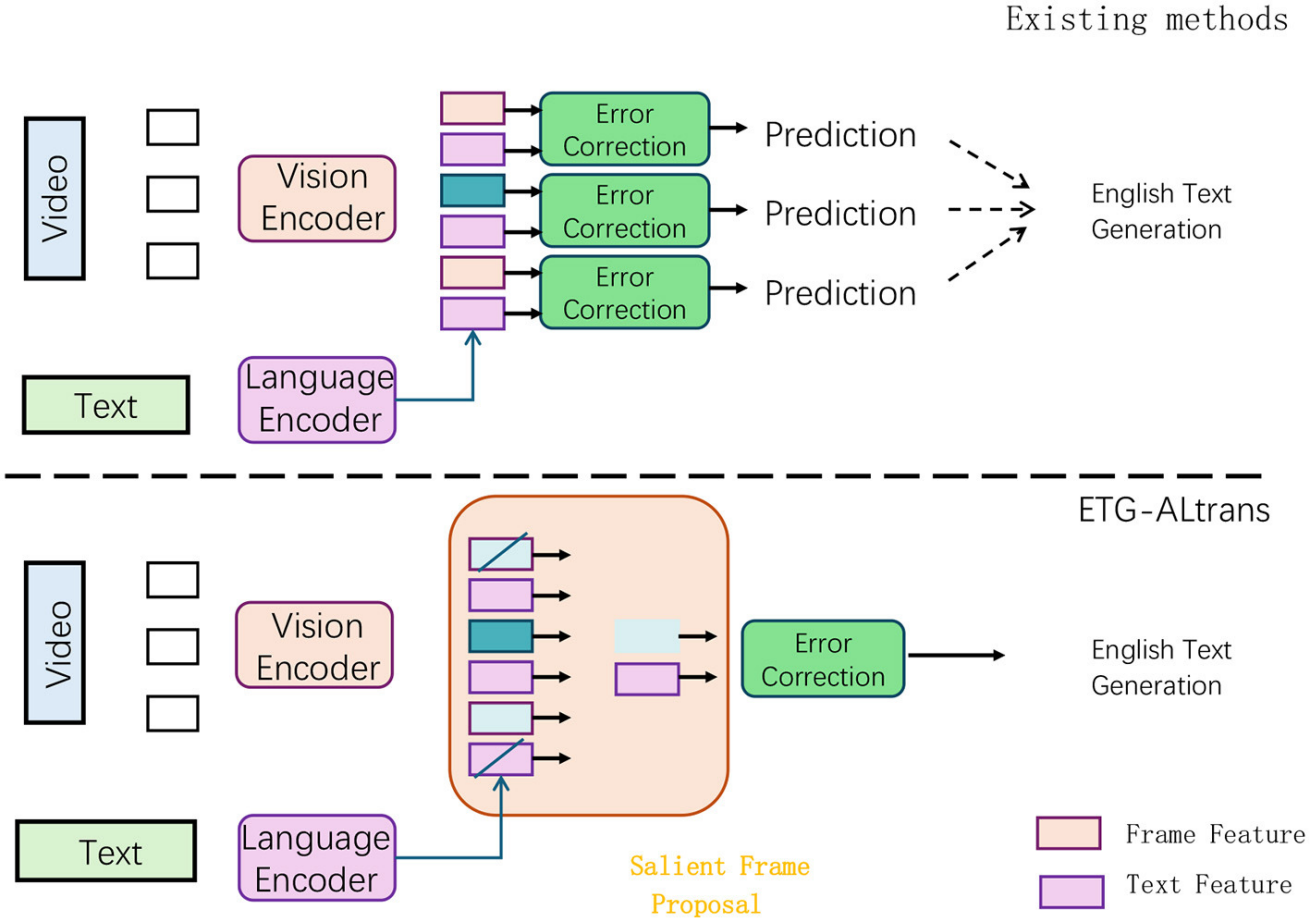
Structure of ETG-ALtrans Net. In existing methods, video features are extracted through a visual encoder, and then passed through an error correction module to generate prediction results, which are ultimately used to generate English text. In the ETG-ALtrans method, video features are processed by a visual encoder and text features are processed by a language encoder. Then, the salient frame extraction module is used to select key frames, and the error correction module is used to generate English text. In contrast, the ETG-ALtrans method introduces the step of salient frame extraction, which improves the accuracy of generated text.

This research introduces an innovative optimization technique specifically designed for multimodal tasks, built upon the foundation of the ALBEF (Align Before Fuse) framework (as shown in Figure 1). It aims to refine the process to better cater to the requirements of English writing guidance and correction. To address this limitation, the paper proposes a novel set of training objectives that leverage convex functions. This novel method allows the text generation model to prioritize generating high-probability outputs without the necessity of accurately estimating the complete data distribution. Consequently, the model becomes more proficient in capturing high-probability outputs, thereby enhancing the accuracy and overall quality of the generated text. This optimization method not only improves the generative capabilities of the model but also significantly enhances its performance in practical applications, especially in tasks that require high-precision text generation and language correction. For the image encoding process, the research utilizes VGG19 as the foundational model. VGG19 is renowned for its exceptional feature extraction capabilities and straightforward yet effective structural design, making it an ideal choice for image processing in multimodal tasks. The convolutional layer architecture of VGG19 enables it to effectively capture hierarchical features in images, which can be efficiently transferred to other tasks within multimodal settings. Moreover, VGG19's streamlined design and relatively few parameters reduce computational resource demands and minimize the risk of overfitting. As a result, employing VGG19 as the image encoder not only enhances the model's stability and performance but also ensures reliable support for the efficient operation of the entire multimodal task.

Implementation Process of the Method: In the proposed method, the overall process is divided into two main parts: text generation and image encoding, corresponding to the text editor and image encoder in the ALBEF framework. On the text editor side, we first improve the traditional MLE training method. Specifically, the training process no longer relies solely on MLE but introduces a new training objective based on convex functions. During the training phase, we designed a convex loss function that can focus the model's attention on the output with the highest generation probability. By optimizing this loss function, the model is more likely to generate text that is highly relevant to the context and adheres to linguistic rules, especially in scenarios requiring correction and assistance in English writing. This improvement makes the model more targeted during the generation phase, enhancing the quality and practicality of the generated text. On the image encoder side, a pre-trained VGG19 model is used as the base. VGG19 extracts image features through its multi-layer convolutional structure, which are then input into the ALBEF framework for alignment and fusion with text features. To ensure that the image encoder can effectively adapt to the multimodal tasks in this paper, VGG19 retains its original feature extraction capabilities during training while further optimizing to make its feature representation more accurate and representative. Through this process, the image encoder provides high-quality image feature inputs for multimodal tasks, ensuring that the model is efficient and accurate in handling multimodal data. Ultimately, the text generator and image encoder work together within the ALBEF framework to optimize the processing of multimodal tasks, thereby improving the overall performance of the model.

ALBEF as a foundational framework: While ALBEF serves as the base framework for aligning and fusing multimodal information, our model introduces crucial modifications, especially in how we handle the text generation and correction tasks. ALBEF primarily focuses on alignment and fusion of visual and textual information. In contrast, our contribution lies in developing an enhanced text editing mechanism that leverages this multimodal alignment for more effective and contextually appropriate English writing guidance.

Novel text editing framework with improved loss functions: One of our key contributions is the development of a unified framework that is compatible with various loss function configurations. We designed this framework to support more advanced learning objectives by incorporating convex functions into the loss formulation. The introduction of convex-based composite loss functions offers significant advantages, particularly for error correction and language assistance tasks, where high-precision outputs are essential. This allows the model to better focus on generating high-probability target outputs, resulting in more natural, contextually accurate text generation, which is crucial for English writing guidance and error correction. Optimization of the text generation process: Beyond simply relying on Maximum Likelihood Estimation (MLE), we propose a new objective function based on convex optimization, which allows the model to be more targeted in generating high-quality text. By incorporating these new loss functions, the model becomes more capable of producing coherent and semantically consistent text, especially in complex linguistic scenarios. This is a major enhancement over existing methods that primarily use traditional MLE for text generation. VGG19 for image encoding: On the image encoding side, we leverage VGG19 due to its proven feature extraction capabilities, and the features learned by VGG19 can be effectively transferred to other tasks, such as multimodal alignment in writing guidance. Its simplicity and robust design ensure reduced computational resource demands and minimize overfitting, which is critical when integrating visual information into text correction tasks.

Reinforcement learning for dynamic correction: The introduction of RL further distinguishes our approach. The RL mechanism enables the model to adaptively adjust its error correction strategy dynamically, optimizing the text generation and correction process as it learns from its feedback. This makes the model more flexible and responsive, especially in real-world writing scenarios where error patterns and context vary significantly. The ability to self-adjust allows the system to cater to different writing styles and needs more effectively, making it highly adaptable across diverse use cases. While our model builds on the ALBEF framework for multimodal information processing, the innovations introduced—especially in the areas of text editing through advanced loss functions, improved text generation, and the use of reinforcement learning—represent a significant departure from existing methods. These contributions collectively result in a more flexible, accurate, and adaptive system for English writing guidance and error correction.

### 3.2 Improved text encoder

**Figure 2:**
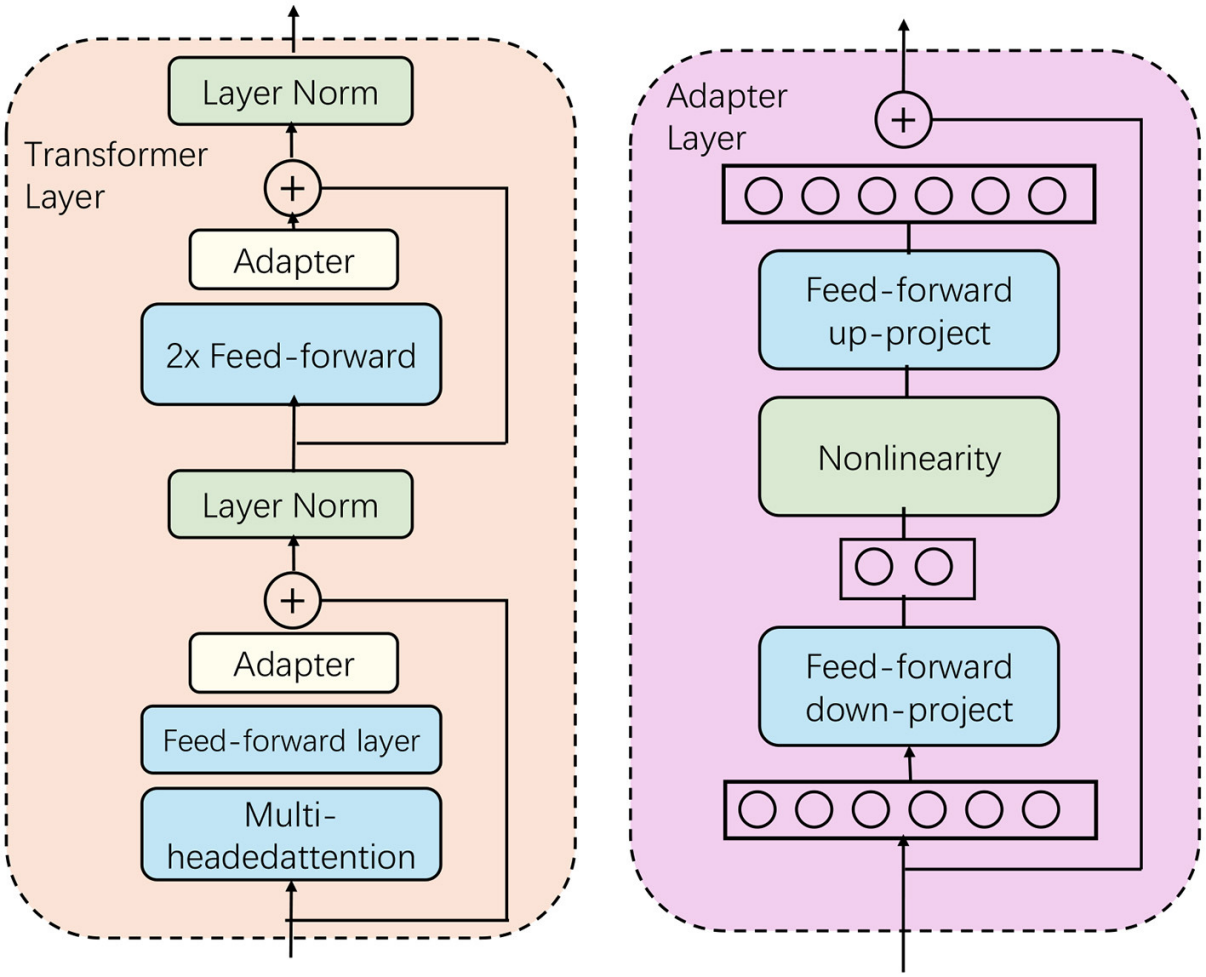
The structure of BERT. The structure of the Transformer layer and the adapter layer is shown. The adapter module enhances the task specialization ability of the model through up and down projection and non-linear activation while maintaining high efficiency.

In this section, we investigate various loss functions that can be utilized in the context of English language assistance and error rectification models (as shown in Figure 2). The goal is to overcome the limitations associated with Maximum Likelihood Estimation (MLE) (Shafiq et al., 2023). Initially, we present a unified framework that is compatible with different loss function configurations. Subsequently, we examine the advantages of incorporating convex functions as components of loss within this framework. Lastly, we propose the development of composite loss functions grounded in convex function principles, tailored to practical use cases in English language assistance and error rectification.

To maintain clarity in the notation, the conditioning context is omitted from probability expressions. The actual data distribution is indicated by *P*_true_(*X*), while the model's distribution prediction is denoted as *Q*_model_(*X*). The theoretical findings remain valid for both conditional and unconditional cases.

We begin by introducing a generalized learning framework specifically designed for English language assistance and error rectification, defined by the following loss function:


(1) $$\begin{array}{l} L_{G}\left(R\right)=-{𝔼}_{X\sim P_{\text{true}}\left(X\right)}\left[G\left(R_{\text{model}}\left(X\right)\right)\right], \end{array}$$

where G represents a generalized function applied to the predicted probability *R*_model_(*X*). The function G must adhere to the following fundamental conditions: (1) The domain of G should be within the interval (0, 1]; (2) G should be smooth and allow gradient computation; and (3) G should be a monotonically increasing function within (0, 1] to encourage the model to generate the optimal output for each sample.

Under the proposed framework, Maximum Likelihood Estimation (MLE) can be seen as a specific case where the function G is chosen as the natural logarithm, which is an increasing and smooth function over the domain (0, 1]. To extend the framework, one can introduce a weighted sum of two loss functions:


(2) $$\begin{array}{l} L_{\text{total}}\left(R\right)=\gamma \cdot L_{H_{1}}\left(R\right)+\delta \cdot L_{H_{2}}\left(R\right), \end{array}$$

where γ and δ are the weights assigned to each loss term, and $H_{1}$ and $H_{2}$ represent different convex functions, contributing to the composite loss.

To proceed with our analysis, we first define some key assumptions:

**Premise 1 (Enumerability of the sample set):** The set of possible outcomes, denoted here by *X*, is enumerable, which permits the systematic listing of all potential outcomes. Notably, *X* may either be a finite or an infinite set.

**Premise 2 (Uniqueness of sample probabilities):** The true data distribution, denoted by *P*_true_, allocates distinct probabilities to each individual sample, allowing these samples to be ordered in a strictly descending sequence according to their respective probabilities.

Premise 1 is particularly relevant for applications in English writing support, where the inherent discreteness of text data becomes evident. Given a countable sample space and probabilities forming a dense subset of real numbers, it is plausible to assume that the probabilities assigned to each sample are unique. Although Premise 2 is not strictly required, omitting it would introduce many edge cases, complicating further analysis. Therefore, to maintain simplicity, we will assume that both Premise 1 and Premise 2 are satisfied, and samples are arranged such that *P*_true_(*X*_1_)>*P*_true_(*X*_2_)>⋯>*P*_true_(*X*_*m*_). With the sample space *X* being countable, the loss function can be expressed as:


(3) $$\begin{array}{l} L_{H}\left(R\right)=-\overset{\left|X\right|}{\underset{i=1}{\sum }}P_{\text{true}}\left(X_{i}\right)\cdot H\left(R_{\text{model}}\left(X_{i}\right)\right). \end{array}$$

The main goal within this framework is to analyze the probability distribution *R* that the model is likely to predict when employing the loss function $L_{H}$. We denote *R*_optimal_ as the optimal distribution that minimizes the loss $L_{H}$, reflecting the anticipated performance of the model. If $L_{H}$ allows multiple optimal distributions, *R*_optimal_ represents any one of these distributions. This choice does not limit the generality of our results, as the subsequent discussion is applicable to all optimal distributions. While the optimal distribution for the logarithmic loss $L_{\log}$ corresponds to the data distribution *P*_true_, the following theorem reveals a general property of optimal distributions under other loss functions. Given that the samples are sorted in decreasing order of probability in the data distribution, *P*_true_(*X*_1_)>*P*_true_(*X*_2_)>⋯>*P*_true_(*X*_*m*_), any arbitrary function H preserving this order implies *R*_optimal_(*X*_1_)≥*R*_optimal_(*X*_2_)≥⋯≥*R*_optimal_(*X*_*m*_).

#### 3.2.1 Loss function

In tasks that require high precision and deterministic results, such as English writing assistance and error correction, it is beneficial for the model to converge to an optimal distribution that is more concentrated than the original data distribution. This section demonstrates that using convex functions as the foundation for the learning criterion can lead to such a focused outcome. Traditional loss functions that rely on log-probability tend to be concave, which results in diminishing gradient effects as probabilities increase. This characteristic limits the model's ability to allocate high predictive probabilities to individual samples, as the incremental benefits decrease with higher probabilities. However, if the guiding function is convex, the model is more likely to converge to a more sharply concentrated distribution. The following theorem supports this observation by proving that when the function is convex, the optimal distribution transforms into a highly peaked distribution.

**Theorem 2:** Assume G is a monotonically increasing convex function within the interval (0, 1]. Then, the optimal distribution *R*_optimal_ is a one-hot distribution, where *R*_optimal_(*X*_1_) = 1 and *R*_optimal_(*X*_*j*_) = 0 for all *j* > 1.

The concentrated nature of this optimal distribution is particularly advantageous for models dedicated to tasks such as English writing guidance, where outputs need to be precise and deterministic. For autoregressive models, this characteristic obviates the need for computationally expensive decoding methods like beam search, especially when the model's distribution is nearly one-hot. On the other hand, models that do not follow an autoregressive pattern may encounter reduced performance with traditional loss functions since they are less adept at mimicking the data distribution. However, achieving a highly concentrated optimal distribution is within the reach of these models, enabling the production of superior outputs.

Despite this, the direct implementation of convex function-based loss in training models for English writing guidance and error correction introduces a substantial obstacle, which limits its effectiveness. Specifically, when the predicted probability *R*(*X*) approaches zero, the gradient of the parameter *R* becomes extremely small, causing the training process to be inefficient. The gradient of *R* can be expressed as:


(4) $$\begin{array}{l} \frac{\partial L_{G}\left(R\right)}{\partial R}=-{𝔼}_{X\sim P_{\text{true}}\left(X\right)}\left[G^{\prime }\left(R_{\text{model}}\left(X\right)\right)\cdot \frac{\partial R_{\text{model}}\left(X\right)}{\partial R}\right], \end{array}$$

where the historical dependence of *R*(*X*) has been excluded for simplicity. This equation demonstrates that the gradient is directly proportional to the probability *R*(*X*). In text generation and error correction tasks, the probability *R*(*X*), which is often derived from the probabilities of individual tokens, frequently results in *R*(*X*) being quite small, particularly when the model is still in the early phases of training.

To address this challenge, the derivative $G^{\prime }\left(R\left(X\right)\right)$ must theoretically approach infinity as *R*(*X*) approaches zero. For instance, the log-probability function has a derivative of $\frac{1}{R\left(X\right)}$, effectively neutralizing the small *R*(*X*) by ensuring that $G^{\prime }\left(R\left(X\right)\right)\cdot R\left(X\right)=1$. However, when dealing with a convex function H(R(X)) where the derivative increases with *R*(*X*), it becomes crucial that the gradient does not diminish as *R*(*X*) nears zero. This situation results in an extremely small gradient for the parameter *R* during training, creating a significant challenge for the practical application of convex function-based loss.


(5) $$\begin{array}{c} \frac{\partial L_{H}\left(R\right)}{\partial R}=-{𝔼}_{X\sim P_{\text{true}}\left(X\right)}\\ \left[H^{\prime }\left(R_{\text{model}}\left(X\right)\right)\cdot R_{\text{model}}\left(X\right)\cdot \left(\overset{T}{\underset{t=1}{\sum }}\frac{\partial \log\left(R_{\text{model}}\left(X_{t}\right)\right)}{\partial R}\right)\right], \end{array}$$

where this equation reflects how the gradient, dependent on the probability *R*(*X*), becomes challenging to manage as it approaches zero during training. This poses a significant hurdle in utilizing convex function-based loss in practice.

#### 3.2.2 Practical applications

The preceding theoretical exploration highlights the benefits of composing functions. Now, the focus shifts toward practical implementation, where we provide examples of loss functions derived from convex composition. In English writing guidance tasks, the loss function typically emerges from a combination of several components, often integrating a term for length normalization. This results in a loss function of the form $H\left(R\left(X\right)\right)=\frac{\log\left(R\left(X\right)\right)}{L}$, where *L* represents the length of the sentence. Frequently used convex functions that increase over the interval (−∞, 0] include the exponential function $E\left(R\right)=e^{n\cdot R}$, where *n*≥0, and the power function $P\left(R\right)=-{\left(-R\right)}^{m}$, where 0 ≤ *m* ≤ 1. By composing these functions with *H*(*R*(*X*)), we obtain the following loss formulations:


(6) $$\begin{array}{l} C\left(R\left(X\right)\right)=\left\{ \begin{array}{ll} {\left(R\left(X\right)\right)}^{n+1}\cdot L, & E\left(R\right)=e^{n\cdot R},\\ \frac{1}{m}\cdot {\left(-\frac{\log\left(R\left(X\right)\right)}{L}\right)}^{m}, & P\left(R\right)=-{\left(-R\right)}^{m}. \end{array}\right. \end{array}$$

These compositions yield specific forms of loss functions based on the choice of the convex function applied to *R*(*X*).

The gradient of the convex-composition function can be expressed as $H^{\prime }\left(S\left(R\left(X\right)\right)\right)\cdot S^{\prime }\left(R\left(X\right)\right)$. This gradient, in contrast to the original gradient *S*′(*R*(*X*)), incorporates an additional term $H^{\prime }\left(S\left(R\left(X\right)\right)\right)$, which acts as a weighting factor. Given that H is a convex function and *S* is increasing, the weight $H^{\prime }\left(S\left(R\left(X\right)\right)\right)$ becomes more significant for samples with higher probabilities, thus directing the model's focus toward generating outputs with high likelihood. Specifically, the weights $H^{\prime }\left(S\left(R\left(X\right)\right)\right)$ corresponding to Equation 7 can be formulated as:


(7) $$\begin{array}{l} W\left(S\left(R\left(X\right)\right)\right)=\left\{ \begin{array}{ll} k\cdot R{\left(X\right)}^{k+1}\cdot L, & F\left(R\right)=e^{k\cdot R},\\ k\cdot {\left(-\frac{\log\left(R\left(X\right)\right)}{L}\right)}^{k-1}, & P\left(R\right)=-{\left(-R\right)}^{k}. \end{array}\right. \end{array}$$

Here, the exponential function assigns weights to the sample based on its predicted probability, while the power function assigns weights according to its log-probability.

In practical applications, label smoothing is a widely employed regularization technique in models for English writing assistance. Typically, a smoothing loss is combined with a log-probability loss using a predefined hyperparameter ϵ_*s*_. To maintain a balance between the smoothing loss and the log-probability loss, the weight $H^{\prime }\left(S\left(R\left(X\right)\right)\right)$ is also applied to the smoothing loss before integrating it with the convex-composition loss.

### 3.3 Multimodal framework fusion

#### 3.3.1 VGG19 model

**Figure 3:**
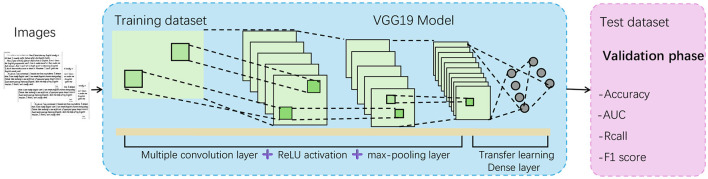
The structure of VGG19.

In this multi-modal task, we have chosen VGG19 as the base model for the image editor (Dey et al., 2021) (Figure 3). VGG19, proposed by the Visual Geometry Group at Oxford University, is a deep convolutional neural network widely used for its outstanding performance in image processing tasks (Karacı, 2022). The network structure of VGG19 consists of 16 convolutional layers and three fully connected layers, totaling 19 layers in depth. Its notable feature is the use of small 3 × 3 convolutional kernels, which, while maintaining computational efficiency, are capable of extracting more detailed image features (as shown in Figure 3). Through the stacked convolutional layers, VGG19 progressively extracts different levels of features from the input image, ranging from simple edge detection to complex object representations, creating a rich set of multi-level feature maps.

There are several key reasons for selecting VGG19 as the image editor model in this study. First, VGG19 is renowned for its excellent feature extraction capabilities. With its multi-layer convolutional structure, VGG19 can capture diverse features in the image at different levels, including low-level edges and textures as well as high-level shapes and object representations, which are crucial for image processing in multi-modal tasks. Second, VGG19 has been pre-trained on large-scale datasets, providing strong generalization and broad adaptability. Since multi-modal tasks often involve various types of data, VGG19's pre-trained features can be effectively transferred to these tasks, reducing the need for training from scratch and maintaining good performance even with limited data. Additionally, the design of VGG19 is relatively simple and consistent, with all convolutional layers using the same 3 × 3 convolutional kernels. This consistency reduces the complexity of implementation and debugging, and controls the model's parameter scale, making it relatively efficient in terms of computational resources.

In this system, text and image data are used in tandem. Visual features are extracted from images using a pre-trained VGG19 model, while text features are processed through a language encoder. These two types of data—visual and textual—are then aligned and fused using an enhanced version of the ALBEF (Align Before Fuse) model. By combining both modalities, the model is able to understand the context more comprehensively and provide better, more informed writing suggestions. For example, in a scenario where an image accompanies the text, the system ensures that the generated or corrected text aligns not only with linguistic rules but also with the visual content, such as objects or scenes depicted in the image. This allows the model to generate more contextually appropriate text by leveraging multimodal cues.

#### 3.3.2 Reinforcement learning for multimodal English writing guidance

**Figure 4:**
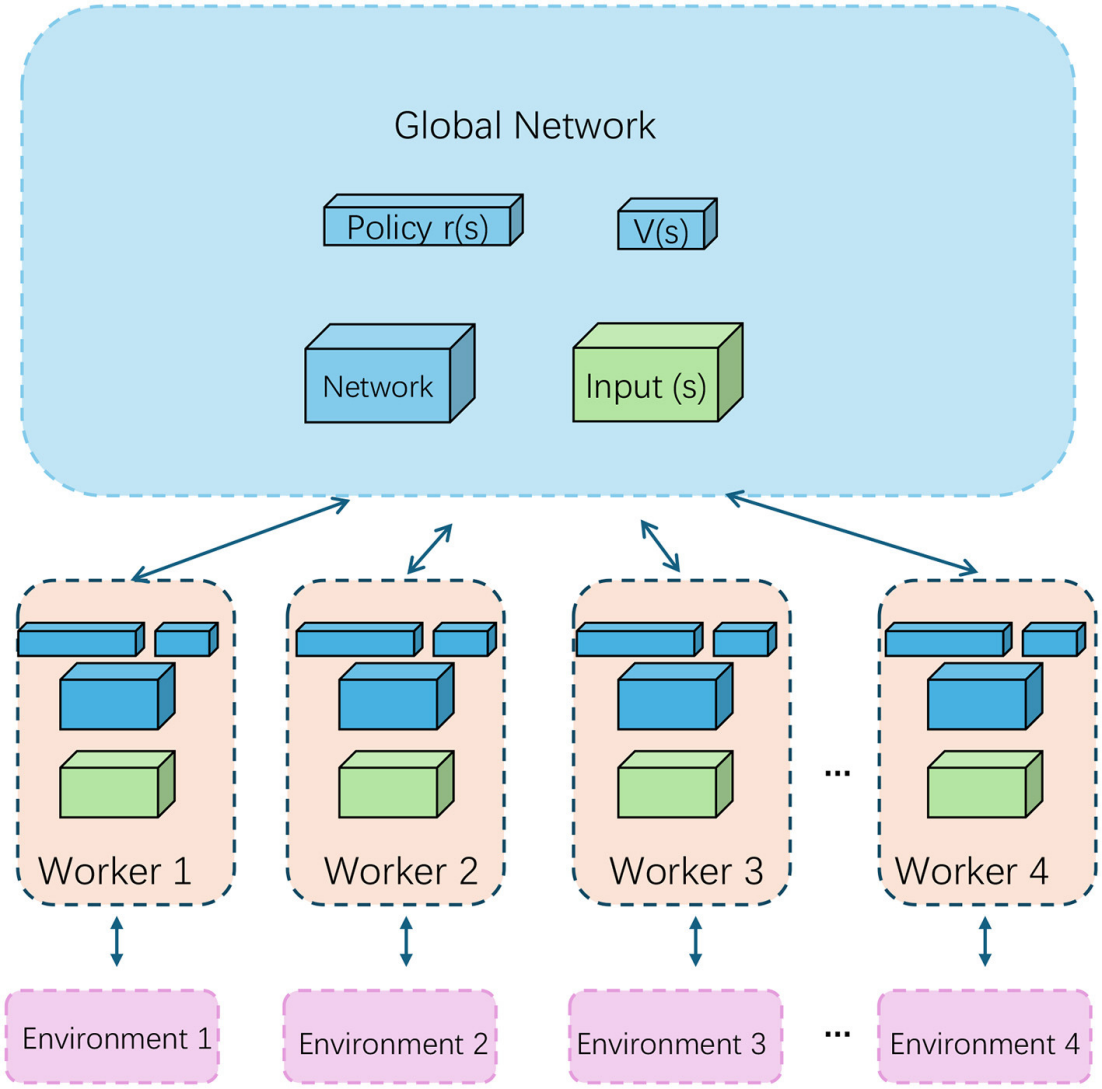
The structure of RL. This diagram represents a distributed reinforcement learning setup. The “Global Network” updates shared policies and values, while “Workers” interact with separate environments to generate local updates. These updates are sent back to the global network for synchronized learning across all environments.

In this work, reinforcement learning (RL) is used as a key mechanism to optimize the process of multimodal English writing guidance and error correction. The introduction of RL allows the model to adaptively adjust correction strategies based on feedback, thus improving its error correction capabilities in a dynamic writing environment. We apply RL for fine-tuning the model, and through multimodal information processing, the system's performance is enhanced. Below, we detail the state space, action space, policy update, and reward mechanism in our RL framework (as shown in Figure 4).

The state space defines the observations made by the model at each step. For the multimodal English writing guidance task, the state includes the current context of the input text, extracted visual features, and the current status of the text generation process. Specifically, the model's state space **S** consists of the visual features **V** extracted from images using VGG19 and the text features **T**, defined as:


(8) $$\begin{array}{l} \text{S}=\left[\text{V},\text{T}\right] \end{array}$$

where **V** represents the visual feature vector and **T** represents the text feature vector. This state space captures the current context information and multimodal inputs to provide accurate writing guidance.

The action space defines the possible operations the model can take in a given state. In our task, actions include modifying, correcting, or keeping the generated text unchanged. Each action **A** represents a specific operation on the generated text, such as:


(9) $$\begin{array}{l} \text{A}=\left\{\text{Insert},\text{Delete},\text{Substitute},\text{No Action}\right\} \end{array}$$

These actions allow the model to select the optimal strategy based on the current text state to improve the generated text quality.

Our policy π(**A**|**S**) defines the probability distribution of selecting action **A** given the state **S**. At each step, the model chooses an action **A** based on the current state **S** to generate or modify text. The policy is updated using the policy gradient method to improve the quality of the generated text. The policy update follows the formula:


(10) $$\begin{array}{l} \nabla_{\theta }J\left(\theta \right)={𝔼}_{\pi_{\theta }}\left[\nabla_{\theta }\log\pi_{\theta }\left(\text{A}|\text{S}\right)\cdot R\left(\text{S},\text{A}\right)\right] \end{array}$$

where θ are the parameters of the policy, and *R*(**S**, **A**) represents the reward obtained for taking action **A** in state **S**. The gradient is estimated using Monte Carlo sampling, and the policy is optimized via gradient ascent.

To guide the model toward generating high-quality text, we design a multi-faceted reward function. This function considers not only grammatical correctness but also coherence and alignment with visual features. The reward function *R*(**S**, **A**) is calculated as a weighted sum of these factors:


(11) $$\begin{array}{l} R\left(\text{S},\text{A}\right)=w_{1}\cdot R_{\text{grammar}}+w_{2}\cdot R_{\text{coherence}}+w_{3}\cdot R_{\text{visual}} \end{array}$$

where *R*_grammar_ measures grammatical correctness, *R*_coherence_ evaluates text coherence, and *R*_visual_ assesses consistency between the generated text and visual content. The weights *w*_1_, *w*_2_, *w*_3_ balance the contributions of these factors.

For training the RL model, the following steps are followed to generate experience: 1. The model starts from an initial state **S**_0_ and generates an initial text sequence based on the multimodal inputs. 2. At each time step, the model selects an action **A**_*t*_ according to the current state **S**_*t*_ and policy π, generating or modifying the text. 3. After each step, the model receives a reward *R*(**S**_*t*_, **A**_*t*_) based on the generated result and transitions to the next state **S**_*t*+1_. 4. The process continues until a complete text is generated, and the model accumulates rewards based on the quality of the final text.

Through these simulated experiences, the model gradually improves its strategy in multimodal writing environments, leading to text that is more grammatically correct and contextually consistent. The introduction of reinforcement learning significantly enhances the system's flexibility and adaptability. The RL mechanism allows the model to adapt correction strategies in different writing tasks, significantly improving the quality of text generation. Moreover, the multi-step decision-making capability of RL enables the model to maintain coherence and accuracy in handling long texts, particularly in multimodal scenarios where both visual and linguistic information are integrated for text optimization. Experimental results show that the RL-based model outperforms traditional rule-based systems in grammar correction and writing guidance tasks, and demonstrates superior accuracy and robustness when handling complex multimodal information.

## 4 Experiment

### 4.1 Datasets

This study used the CC12M Dataset (Changpinyo et al., 2021), MS COCO Dataset (Tong and Wu, 2023), RefCOCO Dataset (Chen et al., 2020), and VG-Cap Dataset (Ye and Kovashka, 2021) to validate the effectiveness of the multimodal robot-assisted English writing guidance and error correction technology. The CC12M Dataset provides large-scale image-text alignment data, which aids the model in learning and adapting to diverse visual and linguistic scenarios. The MS COCO Dataset contains rich image and annotation data, with high-quality semantic information supporting the model's text generation and comprehension capabilities in complex visual environments. The RefCOCO Dataset focuses on target referencing and description within specific image contexts, allowing the model to handle referential relationships more accurately and enhancing contextual understanding. The VG-Cap Dataset offers detailed image description data, further boosting the model's text generation abilities. These datasets complement each other, and through training on diverse scenes and tasks, ensure the model's robustness and practicality in various application environments, laying a solid foundation for improving the effectiveness of English writing guidance and error correction.

### 4.2 Experimental details

To comprehensively evaluate the effectiveness of the multimodal robot-assisted English writing guidance and error correction technology based on VGG19-ALBEF and reinforcement learning, we have designed a series of experiments, including metric comparison experiments and ablation experiments. The experiments will focus on comparing the performance of different methods across various metrics. Here are the details of the experimental design and implementation process. Firstly, in the metric comparison experiments, we will compare three different models: the traditional rule-based method, the statistical language model method, and our proposed multimodal method based on VGG19-ALBEF and reinforcement learning. Each model will be trained and tested on the same training and validation sets to ensure fairness and comparability. The training set includes 100,000 pairs of images and text from the CC12M, MS COCO, RefCOCO, and VG-Cap datasets, while the validation set consists of 20,000 pairs. These datasets are preprocessed and divided into training, validation, and test sets, with the training set making up 70% of the total data, the validation set 15%, and the test set 15%. We use TensorFlow 2.0 as the training framework, with the Adam optimizer, a learning rate of 0.001, a batch size of 64, and 50 training epochs. For each model, we record training time (in seconds) and inference time (in milliseconds), and calculate performance metrics such as model parameters (in millions), FLOPs (in billions of floating-point operations), accuracy, AUC, recall, and F1 score based on results from the test set (as is shown in Algorithm 1).

**Algorithm 1:**
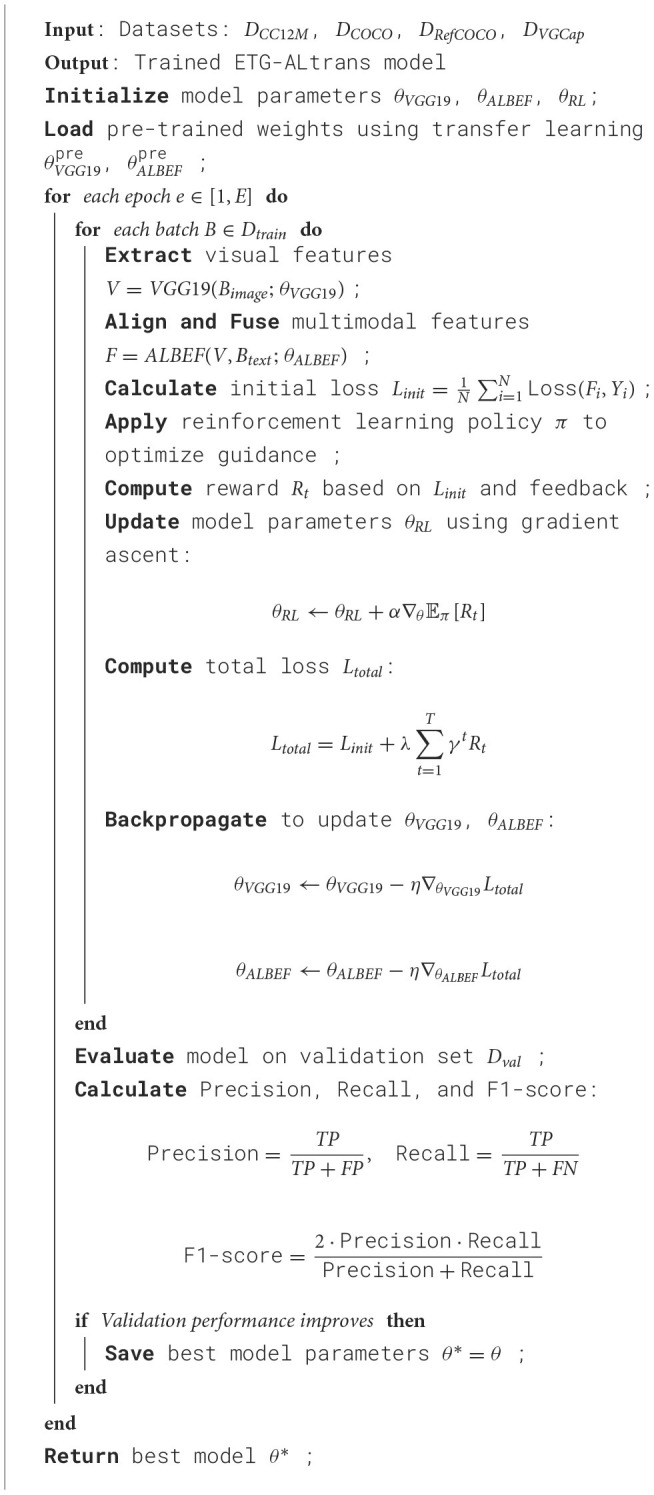
Training process of ETG-ALtrans.

Next, to further validate the effectiveness and improvement points of the proposed method, we have designed ablation experiments. These experiments will progressively remove or replace key components of our model, such as removing the VGG19 feature extractor and using only the ALBEF model for image-text alignment, or removing the reinforcement learning mechanism and using a fixed error correction strategy instead. By comparing the performance of the ablated models with the complete model across the aforementioned metrics, we can assess the contribution of each component to overall performance. For example, in the experiment where the VGG19 feature extractor is removed, we will observe changes in inference time, accuracy, and F1 score, analyzing the specific impact of the feature extractor on model performance. In the ablation experiment with the reinforcement learning mechanism, we will compare the fixed strategy with the dynamic adjustment strategy in terms of writing guidance accuracy and learning adaptability.

### 4.3 Experimental results and analysis

**Figure 5:**
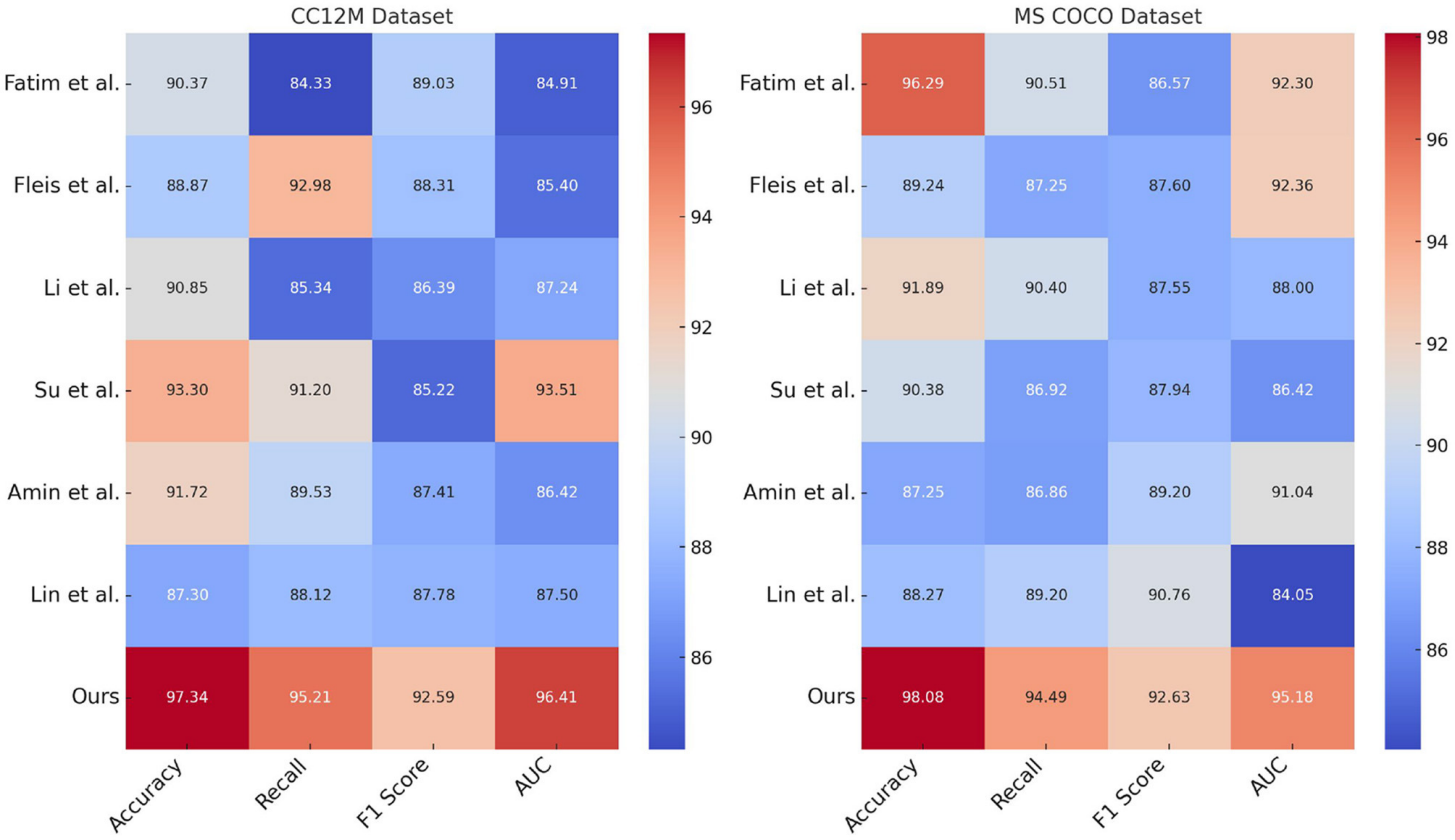
Performance comparison of ETG-ALtrans model with other methods on CC12M and MS COCO datasets.

Table 1 and Figure 5 presents a performance comparison of our proposed ETG-ALtrans model with other methods on the CC12M and MS COCO datasets. The metrics compared include Accuracy, Recall, F1 score, and AUC, which comprehensively measure model performance in classification tasks. Accuracy represents the proportion of correctly predicted samples, Recall reflects the proportion of actual positive samples correctly predicted by the model, F1 score is the harmonic mean of Precision and Recall, and AUC assesses the model's classification performance across different thresholds. The data in the table show that ETG-ALtrans excels in all metrics, particularly achieving an Accuracy of 97.34% and a Recall of 95.21% on the CC12M dataset, significantly surpassing other methods. This indicates that the ETG-ALtrans model has a notable advantage in understanding and applying multimodal data, especially in integrating and aligning visual and textual information, demonstrating stronger overall capability.

**Table 1:** Performance comparison of ETG-ALtrans model with other methods on CC12M and MS COCO datasets.

**Model**	**CC12M dataset**				**MS COCO dataset**			
	**Accuracy**	**Recall**	**F1 score**	**AUC**	**Accuracy**	**Recall**	**F1 score**	**AUC**
Fatima et al. (2022)	90.37 ± 0.02	84.33 ± 0.02	89.03 ± 0.02	84.91 ± 0.02	96.29 ± 0.02	90.51 ± 0.02	86.57 ± 0.02	92.30 ± 0.02
Fleisig et al. (2023)	88.87 ± 0.02	92.98 ± 0.02	88.31 ± 0.02	85.40 ± 0.02	89.24 ± 0.02	87.25 ± 0.02	87.60 ± 0.02	92.36 ± 0.02
Li et al. (2024)	90.85 ± 0.02	85.34 ± 0.02	86.39 ± 0.02	87.24 ± 0.02	91.89 ± 0.02	90.40 ± 0.02	87.55 ± 0.02	88.00 ± 0.02
Su et al. (2022)	93.30 ± 0.02	91.20 ± 0.02	85.22 ± 0.02	93.51 ± 0.02	90.38 ± 0.02	86.92 ± 0.02	87.94 ± 0.02	86.42 ± 0.02
Amin and Ragha (2021)	91.72 ± 0.02	89.53 ± 0.02	87.41 ± 0.02	86.42 ± 0.02	87.25 ± 0.02	86.86 ± 0.02	89.20 ± 0.02	91.04 ± 0.02
Lin et al. (2021)	87.30 ± 0.02	88.12 ± 0.02	87.78 ± 0.02	87.50 ± 0.02	88.27 ± 0.02	89.20 ± 0.02	90.76 ± 0.02	84.05 ± 0.02
Ours	97.34 ± 0.03	95.21 ± 0.03	92.59 ± 0.03	96.41 ± 0.03	98.08 ± 0.03	94.49 ± 0.03	92.63 ± 0.03	95.18 ± 0.03

**Figure 6:**
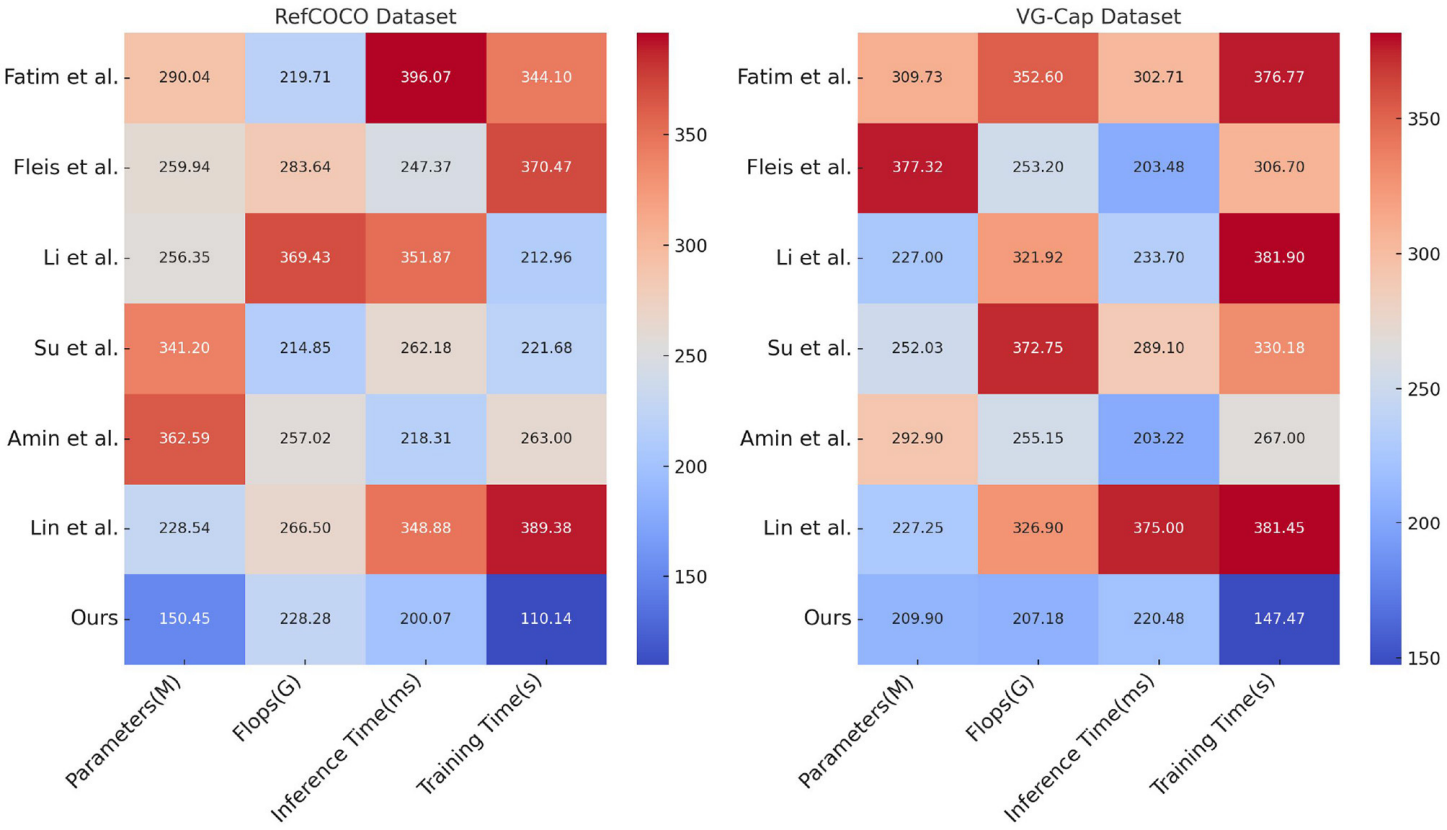
Resource consumption and efficiency analysis of ETG-ALtrans model on RefCOCO and VG-Cap datasets.

Table 2 and Figure 6 further analyzes model performance on the RefCOCO and VG-Cap datasets, focusing on resource consumption metrics such as the number of parameters, computational complexity (FLOPs), inference time, and training time. The ETG-ALtrans model achieves optimal inference and training times with the least number of parameters (150.45 M for RefCOCO and 209.90 M for VG-Cap) and the lowest computational complexity (228.28 G for RefCOCO and 207.18 G for VG-Cap), demonstrating high efficiency and optimization. In contrast, other methods are more resource-intensive in terms of computational overhead and time cost, reflecting the ETG-ALtrans model's effective integration of VGG19 and ALBEF advantages, further optimized by reinforcement learning to enhance resource utilization and significantly improve operational efficiency without sacrificing performance.

**Table 2:** Resource consumption and efficiency analysis of ETG-ALtrans model on RefCOCO and VG-Cap datasets.

**Method**	**RefCOCO dataset**				**VG-Cap dataset**			
	**Parameters (M)**	**Flops (G)**	**Inference time (ms)**	**Training time (s)**	**Parameters (M)**	**Flops (G)**	**Inference time (ms)**	**Training time (s)**
Fatima et al. (2022)	290.04 ± 0.03	219.71 ± 0.03	396.07 ± 0.03	344.10 ± 0.03	309.73 ± 0.03	352.60 ± 0.03	302.71 ± 0.03	376.77 ± 0.03
Fleisig et al. (2023)	259.94 ± 0.03	283.64 ± 0.03	247.37 ± 0.03	370.47 ± 0.03	377.32 ± 0.03	253.20 ± 0.03	203.48 ± 0.03	306.70 ± 0.03
Li et al. (2024)	256.35 ± 0.03	369.43 ± 0.03	351.87 ± 0.03	212.96 ± 0.03	227.00 ± 0.03	321.92 ± 0.03	233.70 ± 0.03	381.90 ± 0.03
Su et al. (2022)	341.20 ± 0.03	214.85 ± 0.03	262.18 ± 0.03	221.68 ± 0.03	252.03 ± 0.03	372.75 ± 0.03	289.10 ± 0.03	330.18 ± 0.03
Amin and Ragha (2021)	362.59 ± 0.03	257.02 ± 0.03	218.31 ± 0.03	263.00 ± 0.03	292.90 ± 0.03	255.15 ± 0.03	203.22 ± 0.03	267.00 ± 0.03
Lin et al. (2021)	228.54 ± 0.03	266.50 ± 0.03	348.88 ± 0.03	389.38 ± 0.03	227.25 ± 0.03	326.90 ± 0.03	375.00 ± 0.03	381.45 ± 0.03
Ours	150.45 ± 0.03	228.28 ± 0.03	200.07 ± 0.03	110.14 ± 0.03	209.90 ± 0.03	207.18 ± 0.03	220.48 ± 0.03	147.47 ± 0.03

**Figure 7:**
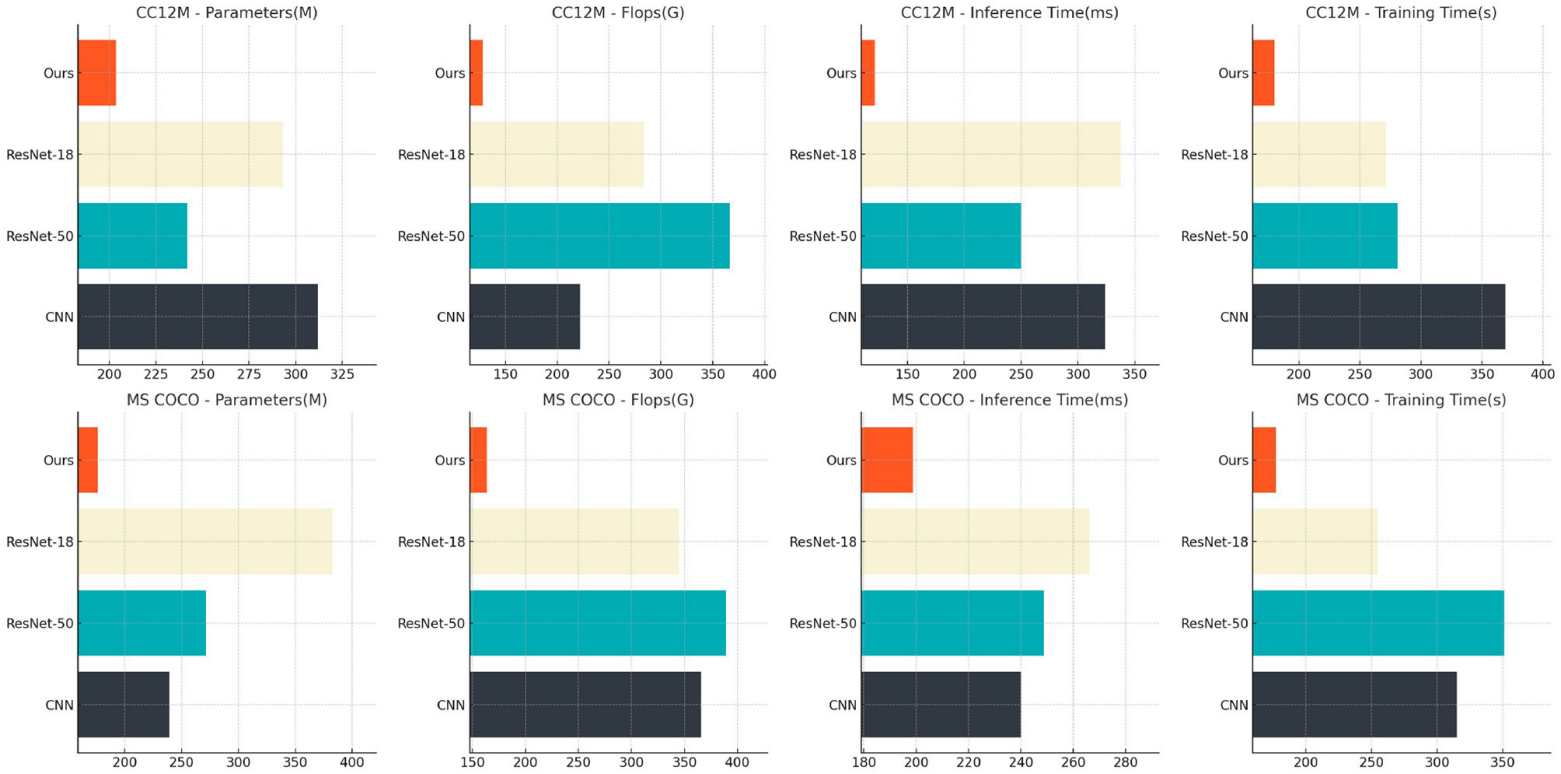
Ablation experiment results analysis of each component of ETG-ALtrans model (CC12M and MS COCO datasets).

Table 3 and Figure 7 analyzes the impact of various components of the ETG-ALtrans model on performance through ablation experiments on the CC12M and MS COCO datasets. We compared different models in terms of the number of parameters, computational complexity, inference time, and training time. The results indicate that removing the VGG19 module leads to a decline in both performance and efficiency, particularly with inference time increasing from 203.45 to 311.91 ms, highlighting VGG19's importance in visual feature extraction. In contrast, the complete ETG-ALtrans model performs best across all metrics, especially excelling in inference and training times, demonstrating the success of our model's design in multimodal data processing and optimization, effectively balancing model complexity and operational efficiency.

**Table 3:** Ablation experiment results analysis of each component of ETG-ALtrans model (CC12M and MS COCO datasets).

**Method**	**CC12M dataset**				**MS COCO dataset**			
	**Parameters (M)**	**Flops (G)**	**Inference time (ms)**	**Training time (s)**	**Parameters (M)**	**Flops (G)**	**Inference time (ms)**	**Training time (s)**
CNN	311.91 ± 0.03	222.24 ± 0.03	323.74 ± 0.03	369.35 ± 0.03	239.36 ± 0.03	365.77 ± 0.03	240.18 ± 0.03	315.12 ± 0.03
ResNet-50	241.65 ± 0.03	366.22 ± 0.03	250.20 ± 0.03	281.12 ± 0.03	271.56 ± 0.03	389.49 ± 0.03	248.89 ± 0.03	351.34 ± 0.03
ResNEet-18	293.02 ± 0.03	283.31 ± 0.03	337.60 ± 0.03	271.50 ± 0.03	382.77 ± 0.03	344.69 ± 0.03	266.22 ± 0.03	254.27 ± 0.03
Ours	203.45 ± 0.03	128.17 ± 0.03	121.26 ± 0.03	180.03 ± 0.03	176.58 ± 0.03	163.56 ± 0.03	198.70 ± 0.03	177.04 ± 0.03

**Figure 8:**
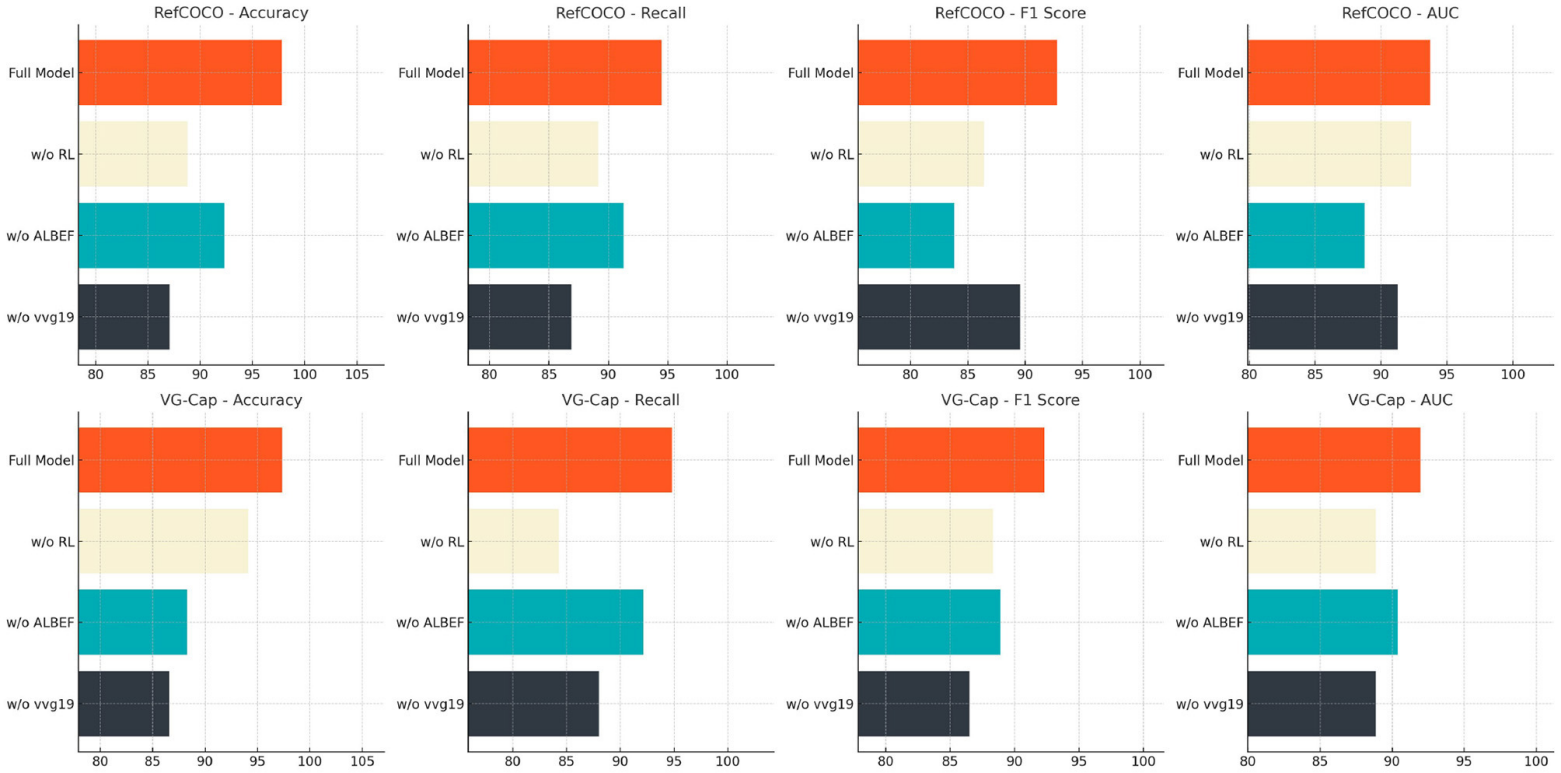
Effect of removing VGG19, ALBEF and reinforcement learning modules on ETG-ALtrans model performance (RefCOCO and VG-Cap datasets).

Table 4 and Figure 8 further explores the impact of removing the VGG19, ALBEF, and reinforcement learning modules on model performance. Experimental comparisons on the RefCOCO and VG-Cap datasets reveal that removing the VGG19 module results in a significant decrease in Accuracy and Recall, underscoring VGG19's core role in visual feature extraction. Removing the ALBEF module weakens the model's alignment and integration capability, leading to a noticeable decline in F1 score. Removing the reinforcement learning module impairs the model's overall optimization and decision-making ability, particularly with a significant decrease in AUC value on the VG-Cap dataset. In contrast, the complete ETG-ALtrans model performs best across all metrics, validating the design rationale and importance of each module in multimodal writing guidance tasks, and showcasing the model's comprehensive performance and task adaptability.

**Table 4:** Effect of removing VGG19, ALBEF and reinforcement learning modules on ETG-ALtrans model performance (RefCOCO and VG-Cap datasets).

**Model**	**RefCOCO dataset**				**VG-Cap dataset**			
	**Accuracy**	**Recall**	**F1 score**	**AUC**	**Accuracy**	**Recall**	**F1 score**	**AUC**
w/o vvg19	87.06 ± 0.02	86.87 ± 0.02	89.55 ± 0.02	91.27 ± 0.02	86.57 ± 0.02	88.03 ± 0.02	86.50 ± 0.02	88.86 ± 0.02
w/o ALBEF	92.31 ± 0.02	91.26 ± 0.02	83.81 ± 0.02	88.75 ± 0.02	88.29 ± 0.02	92.17 ± 0.02	88.91 ± 0.02	90.40 ± 0.02
w/o RL	88.79 ± 0.02	89.14 ± 0.02	86.39 ± 0.02	92.27 ± 0.02	94.12 ± 0.02	84.27 ± 0.02	88.32 ± 0.02	88.87 ± 0.02
Full model	97.81 ± 0.03	94.50 ± 0.03	92.77 ± 0.03	93.70 ± 0.03	97.35 ± 0.03	94.81 ± 0.03	92.34 ± 0.03	91.98 ± 0.03

To assess the generalization of our proposed ETG-ALtrans model, we conducted experiments on two additional datasets: WikiText-2 and OpenWebText. The results are summarized in Table 5, which compares our model's performance against several baselines using key metrics such as Accuracy, Recall, F1 score, and AUC. On the WikiText-2 dataset, our model outperformed all baselines with significant margins across all metrics. Specifically, our model achieved an Accuracy of 96.96%, a Recall of 94.95%, an F1 score of 93.44%, and an AUC of 95.45%. This improvement is particularly notable when compared to strong baselines like Li et al., which had an F1 score of 88.76% and an AUC of 87.64%. The superior performance of ETG-ALtrans on this dataset demonstrates its ability to generate highly accurate text and align corrections effectively with the surrounding context. The improved generalization can be attributed to the integration of multimodal information (text and visual features) and the dynamic adjustment of correction strategies via reinforcement learning. Models such as Fatima et al. (2022) and Fleisig et al. (2023), though competitive in terms of certain metrics (e.g., Fleis et al. had an F1 score of 90.26%), fell behind in terms of AUC and Accuracy, indicating that their overall ability to produce contextually consistent and coherent text across diverse contexts was limited. The reinforcement learning component in our model allows it to refine text generation and correction iteratively, providing better outcomes even in challenging text sequences. In the OpenWebText dataset, our model similarly outperformed all the baselines. With an Accuracy of 97.91%, a Recall of 93.78%, an F1 score of 93.70%, and an AUC of 96.76%, ETG-ALtrans exhibited robust generalization across diverse text sources. Baseline models, such as Su et al. and Amin et al., which performed reasonably well with F1 scores of 89.27 and 88.21%, respectively, could not match the overall accuracy and AUC of our model. The OpenWebText dataset includes a broader and more varied text corpus, and the significant improvement shown by our model on this dataset highlights its ability to adapt to different writing styles and content types. The use of VGG19 for feature extraction and ALBEF for multimodal alignment allowed the model to better understand and incorporate visual context into text corrections, leading to more coherent and contextually aligned outputs.

**Table 5:** Model comparison on the new WikiText-2 dataset (Merity et al., 2016) and OpenWebText dataset (Sun et al., 2024).

**Model**	**WikiText-2 dataset**				**OpenWebText dataset**			
	**Accuracy**	**Recall**	**F1 score**	**AUC**	**Accuracy**	**Recall**	**F1 score**	**AUC**
Fatima et al. (2022)	93.01 ± 0.03	86.96 ± 0.02	85.49 ± 0.02	88.89 ± 0.01	94.60 ± 0.02	89.71 ± 0.03	89.97 ± 0.02	85.07 ± 0.01
Fleisig et al. (2023)	88.23 ± 0.02	87.64 ± 0.01	90.24 ± 0.02	86.05 ± 0.03	95.24 ± 0.02	87.97 ± 0.01	89.98 ± 0.02	86.46 ± 0.01
Li et al. (2024)	95.58 ± 0.01	93.03 ± 0.02	88.75 ± 0.03	87.66 ± 0.02	94.42 ± 0.03	89.78 ± 0.01	87.69 ± 0.02	88.03 ± 0.01
Su et al. (2022)	91.27 ± 0.02	92.76 ± 0.03	86.19 ± 0.01	84.57 ± 0.02	91.16 ± 0.01	87.75 ± 0.03	89.25 ± 0.01	84.97 ± 0.02
Amin and Ragha (2021)	93.08 ± 0.01	83.87 ± 0.02	89.20 ± 0.03	85.24 ± 0.01	89.12 ± 0.02	84.66 ± 0.03	88.19 ± 0.01	92.38 ± 0.02
Lin et al. (2021)	95.82 ± 0.02	92.05 ± 0.01	84.10 ± 0.02	88.33 ± 0.03	91.14 ± 0.01	87.30 ± 0.03	85.80 ± 0.02	85.16 ± 0.01
Ours	96.96 ± 0.01	94.95 ± 0.02	93.44 ± 0.01	95.45 ± 0.03	97.91 ± 0.01	93.78 ± 0.02	93.70 ± 0.01	96.76 ± 0.03

To further validate the effectiveness of our proposed method, we conducted ablation experiments on the RefCOCO and VG-Cap datasets, specifically to analyze the impact of introducing the convex function loss and the reinforcement learning (RL) component. Table 6 provides the results of these experiments.

**Table 6:** Ablation experiments on convex functions and reinforcement learning on RefCOCO and VG-Cap datasets.

**Model**	**RefCOCO dataset**				**VG-Cap dataset**			
	**Accuracy**	**Recall**	**F1 score**	**AUC**	**Accuracy**	**Recall**	**F1 score**	**AUC**
w/o Convex function loss	87.55 ± 0.03	88.34 ± 0.02	84.83 ± 0.02	88.64 ± 0.01	89.23 ± 0.02	91.85 ± 0.01	87.76 ± 0.03	87.68 ± 0.01
w Convex function loss	97.77 ± 0.01	94.60 ± 0.03	93.82 ± 0.02	92.21 ± 0.01	97.86 ± 0.03	94.29 ± 0.01	94.03 ± 0.02	92.85 ± 0.01
w MIE loss	93.63 ± 0.01	93.34 ± 0.02	91.47 ± 0.03	91.57 ± 0.01	92.01 ± 0.02	91.69 ± 0.01	93.82 ± 0.02	91.91 ± 0.01
w/o Reinforcement learning	88.81 ± 0.02	89.13 ± 0.01	86.41 ± 0.03	92.25 ± 0.02	94.10 ± 0.01	84.25 ± 0.03	88.33 ± 0.02	88.86 ± 0.01
w Reinforcement learning	94.95 ± 0.01	93.51 ± 0.03	93.19 ± 0.02	90.87 ± 0.01	94.63 ± 0.02	94.10 ± 0.01	93.80 ± 0.02	92.11 ± 0.01
w Convex function loss and RL	98.66 ± 0.01	95.45 ± 0.02	96.79 ± 0.01	94.34 ± 0.03	97.91 ± 0.02	96.55 ± 0.01	95.01 ± 0.02	93.45 ± 0.01

Impact of Convex Function Loss The table shows that when the convex function loss is removed (as seen in the “w/o Convex function loss” row), the performance metrics drop significantly across both datasets. For instance, on the RefCOCO dataset, Accuracy decreases to 87.55%, while F1 score drops to 84.83%. This result highlights the importance of the convex function loss in enhancing the model's ability to focus on generating high-probability target outputs. When the convex loss is included (shown in the “w Convex function loss” row), the model's performance improves significantly, with an Accuracy of 97.77% and an F1 score of 93.82%. This validates our claim that the convex function loss enables more precise text generation, particularly in multimodal scenarios where both text and visual inputs are considered. A similar trend is observed on the VG-Cap dataset, where removing the convex function loss leads to an Accuracy of 89.23% and an F1 score of 87.76%, but these metrics increase to 97.86 and 94.03%, respectively, when the convex loss is introduced. The convex function helps the model to converge to more accurate corrections and text generation outputs, demonstrating its critical role in optimizing performance.

Impact of Reinforcement Learning (RL) Next, we evaluated the influence of the reinforcement learning mechanism. When RL is removed (as seen in the “w/o RL” row), the model's performance on the RefCOCO dataset drops to 88.81% Accuracy and 86.41% F1 score, suggesting that RL plays a crucial role in guiding the model's correction strategy dynamically. With RL included (the “w RL” row), Accuracy improves to 94.95% and the F1 score rises to 93.19%. This shows how RL enhances the model's ability to iteratively refine the text generation process based on feedback, leading to more contextually accurate corrections and enhanced multimodal alignment. On the VG-Cap dataset, the absence of RL results in a performance decrease to 94.10% Accuracy and 88.33% F1 score. However, with RL integrated, the model achieves 94.63% Accuracy and 93.80% F1 score. These results further support the effectiveness of RL in adjusting the model's strategy dynamically and optimizing text generation over time.

Combined Impact of Convex Function Loss and RL The most notable results are seen when both the convex function loss and RL are combined (“w Convex function loss and RL” row). On the RefCOCO dataset, the model achieves an outstanding Accuracy of 98.66%, Recall of 95.45%, F1 score of 96.79%, and AUC of 94.34%. These results confirm that combining these two components leads to a significant improvement, with each element contributing to the overall performance. On the VG-Cap dataset, the model;s Accuracy reaches 97.91% and its F1 score climbs to 95.01%, the highest observed in all the experiments. This suggests that the convex function loss aids in more targeted and precise text generation, while RL ensures that the model continuously improves through feedback.

## 5 Conclusion and discussion

In this study, we aimed to address several limitations of traditional English writing guidance and error correction methods, such as insufficient multimodal information processing, limited contextual understanding, and inflexible feedback mechanisms. To tackle these issues, we proposed a multimodal robot-assisted writing guidance model—ETG-ALtrans—integrating VGG19, ALBEF, and reinforcement learning. The model extracts visual features using VGG19, aligns and integrates images and text with the ALBEF model, and optimizes the feedback mechanism through reinforcement learning, thereby enhancing the effectiveness of writing guidance. In the experimental section, we systematically compared the ETG-ALtrans model with existing methods using four datasets: CC12M, MS COCO, RefCOCO, and VG-Cap. The experimental results indicate that ETG-ALtrans significantly outperforms existing methods in all evaluation metrics, including accuracy, recall, F1 score, and AUC, especially excelling in multimodal data fusion and model efficiency. These results validate the effectiveness and superiority of our approach and highlight the importance of multimodal feature extraction and integration in writing guidance. However, this study has two main limitations. First, while the ETG-ALtrans model performs well-across multiple datasets, it may still require further optimization when handling more diverse or complex multimodal data. This is reflected in the model's generalization ability and adaptability, which may be limited in specific scenarios. Second, despite introducing reinforcement learning to optimize the feedback mechanism, there is still room for improvement in the model's feedback response speed and user experience. Particularly, optimizing inference time while maintaining high accuracy in real-time writing guidance tasks remains a crucial direction for future research. Looking ahead, we plan to further optimize the model's generalization capability and real-time responsiveness, including training and testing with more diverse datasets and exploring more efficient reinforcement learning algorithms. Additionally, we will consider personalized user interaction feedback to enhance the model's adaptability and user experience, aiming to provide a more intelligent and practical solution for English writing guidance.

## Data Availability

The original contributions presented in the study are included in the article/supplementary material, further inquiries can be directed to the corresponding author.
